# Post-traumatic stress disorder symptoms following exposure to acute psychological trauma in children aged 8–16 years in South Africa: protocol for the Sinethemba longitudinal study

**DOI:** 10.1136/bmjopen-2024-085129

**Published:** 2024-07-11

**Authors:** Tamsin H Sharp, Yeukai Chideya, Alessandra Giuliani, Xanthe Hunt, Mark Tomlinson, Soraya Seedat, Cathy Creswell, Pasco Fearon, Catherine Hamilton-Giachritsis, Rachel Hiller, Richard Meiser-Stedman, Stefani Du Toit, Jackie Stewart, Sarah L Halligan

**Affiliations:** 1 Department of Psychology, University of Bath, Bath, UK; 2 Institute for Life Course Health Research, Department of Global Health, Stellenbosch University, Stellenbosch, South Africa; 3 School of Nursing and Midwifery, Queen's University Belfast, Belfast, UK; 4 Department of Psychiatry, Stellenbosch University, Stellenbosch, Western Cape, South Africa; 5 Department of Psychology, Stellenbosch University, Stellenbosch, Western Cape, South Africa; 6 Department of Experimental Psychology, University of Oxford, Oxford, UK; 7 University of Cambridge Centre for Family Research, Cambridge, UK; 8 Division of Psychology and Language Sciences, University College London, London, UK; 9 Department of Clinical Psychology and Psychological Therapies, University of East Anglia Norwich Medical School, Norwich, UK; 10 Department of Psychiatry, University of Cape Town, Rondebosch, South Africa; 11 Department of Global Health, Stellenbosch University, Stellenbosch, South Africa; 12 Department of Surgery, University of Cape Town, Rondebosch, South Africa

**Keywords:** EPIDEMIOLOGIC STUDIES, MENTAL HEALTH, PAEDIATRICS, Child & adolescent psychiatry, PUBLIC HEALTH, Psychological Stress

## Abstract

**Abstract:**

**Introduction:**

Children exposed to trauma are vulnerable to developing post-traumatic stress disorder (PTSD) and other adverse mental health outcomes. In low-and middle-income countries (LMICs), children are at increased risk of exposure to severe trauma and co-occurring adversities. However, relative to high-income countries, there is limited evidence of the factors that predict good versus poor psychological recovery following trauma exposure in LMIC children, and the role of caregiver support in these high-adversity communities.

**Methods and analysis:**

We will conduct a longitudinal, observational study of 250 children aged 8–16 years and their caregivers in South Africa, following child exposure to acute trauma. Dyads will be recruited from community hospitals following a potentially traumatic event, such as a motor vehicle accident or assault. Potential participants will be identified during their hospital visit, and if they agree, will subsequently be contacted by study researchers. Assessments will take place within 4 weeks of the traumatic event, with 3-month and 6-month follow-up assessments. Participants will provide a narrative description of the traumatic event and complete questionnaires designed to give information about social and psychological risk factors. Child PTSD symptoms will be the primary outcome, and wider trauma-related mental health (depression, anxiety, behavioural problems) will be secondary outcomes. Regression-based methods will be used to examine the association of psychosocial factors in the acute phase following trauma, including caregiver support and responding, with child PTSD and wider mental health outcomes.

**Ethics and dissemination:**

Ethical approvals have been granted by Stellenbosch University and the University of Bath, with additional approvals to recruit via hospitals and healthcare clinics being granted by the University of Cape Town, the Department of Health and the City of Cape Town. Study findings will be disseminated via publication in journals, workshops for practitioners and policy-makers, and public engagement events.

**STRENGTHS AND LIMITATIONS OF THIS STUDY**

- Longitudinal methods were piloted in settlement communities of Cape Town, enabling strong links to be established with proposed recruitment sites.
- Focus groups have been conducted with members of the community to ensure proposed study methods and materials are culturally sensitive.
- Pilot work has demonstrated excellent retention rates, however, some missing data will be expected due to study participant attrition. This will be managed using multiple imputation methods during analysis.
- A limited set of biological samples will be collected, including heart rate data and dried blood spot samples. This will allow a robust examination of the biological predictors of childhood post-traumatic stress disorder while maximising study acceptability.

## Introduction

 Periurban communities in South Africa are characterised by extreme psychosocial adversity and epidemiological research reports high levels of child exposure to traumatic events.^1 2^ Such communities are also environments where children experience chronic and often profound adversity.^3 4^ One such community is Khayelitsha, Cape Town, where the majority of the approximately one million inhabitants live in makeshift housing, with widespread poverty and extremely high levels of violent crime.^5 6^ Rates of trauma exposure in this community are above 80%,^3 7^ including frequent exposure to severe interpersonal violence (eg, half of 8–13 years report witnessing murder).^8^ Preliminary work in this community indicates a significant proportion of children are exposed to multiple and severe traumas.^9 10^ Such exposure may impact child’s psychological well-being and may be associated with a range of adverse outcomes, with implications for broader social adjustment, educational attainment and physical health.

One psychological outcome for trauma-exposed children is the development of post-traumatic stress disorder (PTSD). The point prevalence of youth PTSD in high-risk South African communities is estimated at 20%–30%, which is markedly high.^3^ Consistent with this, in screening young people recruited from schools in Khayelitsha, we found a prevalence of probable PTSD of 28%.^11^ PTSD in children is a highly persistent problem,^12 13^ associated with serious comorbid psychological difficulties, including depression, conduct problems and substance use, as well as poorer criminal justice outcomes.^14^,^17^ PTSD can also have a significant impact on social, emotional and educational outcomes, and thus presents a significant threat to a young person’s developmental trajectory.

Despite the high prevalence of trauma and the consequences of developing PTSD, there is little evidence relating to profiles of acute psychological recovery post-trauma in children from high-risk communities. Available longitudinal evidence derives almost exclusively from high-income country (HIC).^18^ While studies of lower-risk samples show children typically have acute elevations in PTSD symptoms post-trauma, followed by significant rates of spontaneous recovery over 3–6 months,^18^ it is unclear whether this applies in high-risk contexts. This includes settings of severe and chronic adversity such as South Africa, where historical trauma and ongoing trauma are pervasive.^7^ Obtaining information regarding longitudinal profiles of psychological recovery in low-income and middle-income country (LMIC) following trauma is critical to developing appropriate research and intervention strategies.

In addition to limited knowledge of the longitudinal profiles of psychological recovery posttrauma in LMIC children, most of our understanding of the factors that predict better or worse outcomes is also based on HIC populations. First, there is robust evidence that individual psychological responses to trauma, specifically negative trauma-related appraisals, disorganised and highly sensory trauma memories, and maladaptive coping behaviours, predict children’s persistent distress.^19^ Second, there is increasing evidence that quality of post-trauma support children receive from caregivers can play a role in their recovery.^20^ Third, there is some evidence that biological stress responses, including autonomic reactivity,^21^ hypothalamic–pituitary–adrenal axis function,^22 23^ and inflammatory response^24^ may be associated with children’s PTSD, but these components have rarely been studied longitudinally. In addition, there is emerging evidence that childhood adversity is associated with poorer physical health outcomes in adulthood, such as cardiovascular disease.^25^ Across all proposed protective or risk factors for child recovery after trauma, equivalent evidence from high adversity populations of children is lacking.

To address this, we aim to conduct a robust examination of the psychosocial predictors of children’s mental health outcomes following trauma exposure among families growing up in high adversity communities in Cape Town, South Africa, with a specific focus on the association between caregiver factors and child recovery. In addition, we will examine a limited set of biological predictors, including DNA methylation profiles and autonomic reactivity. Through this large-scale, longitudinal investigation of children and caregivers in the 6 months following trauma, we will gain systematic information about pathways to recovery versus persistent PTSD in children in a high adversity context.

## Aims and objectives

The Sinethemba study will allow for a robust assessment of the social, psyschological and biological predictors of child PTSD and mental health symptoms after trauma, addressing the following objectives:

1. To examine trauma-related aspects of caregiver support as predictors of initial child PTSS post-trauma and symptom change over time, with child cognitive-behavioural processes as potential mediating variables.
2. To examine pathways from caregiver trauma and PTSD to child outcomes, via caregiver responding.
3. To test whether predictors of child PTSS also predict wider aspects of post-trauma mental health, with child cognitive-behavioural processes as potential mediating variables.
4. To obtain longitudinal information about the risk of PTSD and the extent of recovery in the acute phase post-trauma in a high adversity, LMIC population.
5. To identify biological predictors of children’s recovery versus persistent PTSD, including through measurement of the autonomic nervous system and epigenetic markers.

## Methods

### Study design and population

The Sinethemba study will comprise a prospective longitudinal examination of trauma-exposed children and their caregivers, conducted in Cape Town, South Africa, which incorporates detailed assessments of caregiver support as well as measures of child PTSD symptoms. The study was named by residents of Khayelitsha who are involved in data collection, referring to the isiXhosa phrase ‘we have hope’. Study recruitment will take place from August 2022 to April 2024.

We will recruit 250 children, aged 8–16 years, and their primary caregivers via community hospitals following child exposure to acute trauma in Khayelitsha and surrounding areas of South Africa. Khayelitsha contains both formal settlements (government housing with onsite water and sewage) and informal settlements (shacks or temporary structures that rarely have water or access to sanitation on the premises).

Participants will be recruited via healthcare facilities following the child’s involvement in a potentially frightening or psychologically traumatic event (eg, assault, motor vehicle accident, serious accidental injury). Specific recruitment sites included Khayelitsha District Hospital, Tygerberg Hospital, Red Cross Hospital, Matthew Goniwe Clinic, Kuyasa clinic, Town Two Clinic and Site B Youth Clinic. Families who present at the facility for this reason will be informed about the study by a member of the research team. A brief outline of the study will be provided to the caregiver at this stage, and potential participants will receive a study information leaflet. If a caregiver expresses interest, the recruiting staff member will take down the caregiver’s contact details and provide these details to the research team. The research team will subsequently contact those who expressed an interest, provide them with more detailed information and invite them to participate. Children will be excluded based on the following criteria: intellectual disability that precludes mainstream schooling, a history of organic brain damage, presenting with current self-harm or active suicidal intent, trauma inflicted by the primary caregiver or the child being under child protective services. In addition, child–caregiver dyads will be excluded if they do not speak isiXhosha or English.

### Measures

Caregivers and children will participate in a trauma recall task,^26^ alongside answering questionnaires. In addition, children will have the option of providing biological data. Measures are summarised in table 1 and detailed below. Assessments will be administered in isiXhosa or English, and at every time point unless otherwise specified.

**Table 1:** Description of the measures collected for the Sinethemba study

Measure	Indexes	Time points
Caregiver report		
Background interview/hospital record form	Measuring demographics/background information, trauma characteristics	T1
Parental Trauma Response Questionnaire	Measuring caregiver responses to child trauma	T1, T2, T3
Post-traumatic Diagnostic Scale	Measuring caregiver PTSD	T1, T2, T3
Self-Reporting Questionnaire	Measuring caregiver depression and anxiety	T1, T2, T3
Faith-based coping	Individual items of faith-based coping	T1, T2, T3
Modified Background Interview	Measuring changes in demographics, ongoing trauma-related problems	T2, T3
Pubertal Development Scale	Assess the development of puberty	T1, T3
Child PTSD Symptom Scale–parent report (CPSS)/UCLA Trauma Screen	Measuring child trauma, PTSD symptoms and diagnosis	T1, T2, T3
Child Behaviour Checklist (CBCL)	Measuring child mental health	T1, T2, T3
Child report		
CPSS self-report/UCLA Trauma Screen	Measuring child trauma and PTSD	T1, T2, T3
The Children’s Revised Impact of Event Scale	Measuring child potential pre-existing PTSD symptoms and diagnosis	T1
Trauma Memory Quality Questionnaire	Measuring child trauma memory characteristics	T1, T2, T3
Child Posttraumatic Cognitions Inventory	Measuring child trauma-related cognitions	T1, T2, T3
Child Posttrauma Coping Questionnaire	Measuring child posttrauma coping responses	T1, T2, T3
International Trauma Questionnaire–Child and Adolescent Version	Measuring complex PTSD	T1, T2, T3
Kidcope	Measuring child social support	T1, T2, T3
Faith-based coping	Individual items of faith-based coping	T1, T2, T3
Revised Children’s Anxiety and Depression Scale–Short form	Measuring child anxiety and depression symptoms	T1, T2, T3
YSR/CBCL-Rule breaking scale	Measuring child conduct problems/rule breaking	T1, T2, T3
Heart rate monitoring	Measuring child autonomic function	T1, T2, T3
Substance use	Measuring alcohol and tobacco use	T1, T2, T3
Dried blood spots	Measure biomarkers including DNA methylation	T1, T2, T3
Anthropometric measures	Measuring height and weight to measure physical development	T1, T2, T3
Stop-distance assessment	Measuring comfortable interpersonal distance	T3
Narrative tasks		
Caregiver narrative	Measuring caregiver trauma-related appraisals and support	T1
Child narrative	Measuring child trauma-related appraisals and support	T1

PTSDpost-traumatic stress disorderUCLAUniversity of California at Los AngelesYSRYouth self-report

### Background characteristics

Demographic factors will be indexed via an initial background interview and a hospital record form, collected at the baseline assessment only. The hospital record form will collect data relating to the child on admission to hospital (temperature, heart rate, respiration rate and level of consciousness), when available. At follow-up assessments, a background interview will be completed, asking about any major changes in circumstances and the child’s current physical and mental health.

### Child-reported measures

#### Psychosocial processes

1. Children will participate in a trauma recall task, where they will be asked to narratively describe the index traumatic event in as much detail as they can.^26^ When the child has finished describing the event, the data collector will ask additional questions to capture details regarding how the child feels they and their caregiver have coped since the traumatic event. Transcripts will be coded for the degree of memory disorganisation and sensory/emotional qualities, and negative/positive trauma-related appraisals, using established methodology.^27 28^
2. The adapted Trauma Memory Quality Questionnaire will capture the child’s own perceptions of their memories of the event (disorganisation/sensory-perceptual features).^29^
3. The Child Post-Traumatic Cognitions Inventory^30^ will assess appraisals relating to the world as dangerous/the self as vulnerable, and to trauma sequelae as being indicative of permanent and disturbing change.
4. Child post-trauma coping will be captured via the Child Post-trauma Coping Questionnaire, which indexes a range of coping behaviours (eg, rumination, dissociation, avoidance)^20^; items on faith-based coping, developed in collaboration with a local advisory board (included based on qualitative work with the study population); and the Kidcope scale, which measures engagement with social support post-trauma.^31^
5. The UCLA (University of California at Los Angeles) Trauma Screen will be used with the Child Revised Impact of Events Scale-8 to measure previous trauma exposure and pre-existing PTSD symptoms^32^ at baseline. At follow-up time points, only the trauma screen will be readministered to capture any new trauma exposures.
6. A stop-distance task, capturing children’s willingness to physically approach an unfamiliar adult, will be used to assess children’s comfortable interpersonal distance, which has previously been identified as a fundamental social process that is associated with trauma (ie, larger interpersonal distances preferred in trauma exposed children).^33 34^ This task will be completed at T3 only.

#### Psychosocial outcomes

1. The Child PTSD Symptom Scale (CPSS) will measure PTSD symptoms following the index traumatic event,^35^ with additional items from the International Trauma Questionnaire included to measure complex PTSD.^36^
2. The Revised Children’s Anxiety and Depression Scale-short form will measure symptoms of anxiety and depression.^37^
3. The Youth Self-Report Rule Breaking Scale will measure rule breaking and risky behaviour,^38^ with additional items included to measure alcohol and tobacco use.

#### Caregiver-reported measures

1. Caregivers will also participate in a trauma recall task,^26^ with prompts used to ensure that beliefs relating to the causes and consequences of the trauma and ways of coping are considered. Transcripts of narratives will be used to generate indices capturing the extent of caregiver threat appraisals/threat enhancement and encouragement versus discouragement of maladaptive coping styles in the child, using established methodology.^20^
2. The Post-traumatic Diagnostic Scale will be used to measure caregiver’s PTSD symptoms,^39^ and the Self Reporting Questionnaire used to index depression and anxiety symptoms.^40^
3. The Parent Trauma Response Questionnaire will be used to capture the extent to which caregivers provide appraisals that enhance threat perceptions posttrauma, and their facilitation of child coping styles (eg, avoidance of trauma talk or behavioural reminders), including additional faith-based coping items.^41^
4. The CPSS–Parent report^39^ and the Child Behaviour Checklist will be used to measure the caregiver’s perception of their child’s trauma and mental health symptoms.^38^

### Biological measures

#### Dried blood spots

Blood samples will be collected from children to measure genome-wide DNA methylation. Samples will be collected by applying a few drops of blood, drawn by lancet from a finger, onto absorbent filter paper. Samples will be stored in a secure freezer at −80°C at Stellenbosch University. DNA will be extracted from each of the samples, which will be analysed using DNA methylation microarray (Illumina EPIC BeadChip) at USC Molecular Genomics Core.

#### Heart rate measurement

Heart rate will be monitored in children during a 5 min baseline period and during the trauma recall task. ECG monitoring provides an index of sympathetic arousal (heart rate change), as well as of parasympathetic regulation (heart rate variability) and will be used to address biological models of PTSD that assume hyperactivity in the autonomic nervous system post-trauma. Heart rate will be monitored in children using the PLUX Biosignals system (https://www.pluxbiosignals.com/). Data will be recorded directly using OpenSignals revolution software (https://support.pluxbiosignals.com/knowledge-base/introducing-opensignals-revolution/). Cardiac biomarkers will be extracted from ECG recordings using ANSLAB software (https://www.anslab.net/wp/).^42^

### Anthropometric measures

Weight and height of children will be measured, allowing us to track physical development over the course of the study. In addition, the Pubertal Development Scale will be used to assess the child stage of puberty.^43^ Based on feedback from the study team, the self-report form will be used for children aged 13 years and above, and the parent report for those who are younger.

### Procedure

Participants will be assessed 2–4 weeks post-trauma (T1), with follow-up assessments 3 months (T2) and 6 months (T3) later. Caregivers and children will be assessed separately, including questionnaires, interviews, tasks and biological measurements for children (heart rate monitoring, measures of height and weight and dried blood spot (DBS) samples), as detailed under meausures. With consent, basic data relating to hospital admission for the index event will be extracted from participants’ medical records.

Data collection will take place at the Masipuhlisane Research Centre in Khayelitsha or at participants’ homes, depending on participant preference. Researchers at the centre are highly experienced in conducting data collection with vulnerable groups such as at-risk young people, including research surrounding sensitive topics such as trauma. Participants will be provided with transport to and from the centre. Follow-up assessments may be conducted over the telephone, with an option of an abbreviated questionnaire containing primary measures only, if participants have relocated. These options will be useful in establishing optimal follow-up procedures and may increase overall retention rates.

In order to ensure that participants feel comfortable to answer as openly as possible, caregivers and children will be interviewed separately. Caregivers will complete a background information interview and a set of questionnaires while children will complete separate questionnaires. Caregivers and children will additionally be asked to provide narrative descriptions of the index traumatic event (ie, the event that led to the child’s recent hospital visit). In addition, children will be invited to provide biological assessments.

### Data management

Data will be collected using ODK (https://getodk.org/), a secure digital system which allows researchers in different locations to access the same datasets. Data will be held at Stellenbosch University and the University of Bath, with digital data stored on central servers which are backed up daily. Digital files will be stored by numeric identifier, with personal details being kept separately. Consent forms and paper documentation will be stored in locked cabinets at the study site, accessible only to study staff.

### Sample size and power calculation

The study is powered based on detecting longitudinal associations between caregiver responding and child PTSS. A sample size of 200 is required to detect an effect size for the association between social predictors and follow-up PTSD symptoms equivalent to an R^2^=0.03 with 80% power at α=0.05, assuming that baseline variables account for an R^2^ of 0.23 and that five predictors are included in the model. We will be able to detect effects of R^2^=0.04 or greater with 90% power. Estimates of effects are based on the outcomes of our equivalent UK study, where longitudinal effect sizes for parenting variables ranged from R^2^=0.10 to R^2^=0.03, controlling for initial child symptoms and other key covariates, or from R^2^=0.18 to R^2^=0.04 when initial symptoms were not controlled for.^20^ For biological predictors, there is limited evidence to inform a sample size calculation. However, given a sample size of 200, we will have 81% power to detect a 0.2 SD change in the outcome (eg, PTSS score) per 1 SD change of an independent variable (eg, heart rate), assuming adjustment for covariates. To put this in context, a 0.2 SD in heart rate is 2.8 bpm or <1 mg/L of CRP (based cohort data for 14 years).^44^ Relevant longitudinal correlations in the limited existing literature range from 0.18 (for HR)^21^ to 0.31 for cortisol.^45^

Pilot work has achieved 100% follow-up rates and this excellent retention is consistent with previous work. A recent longitudinal study from the same community achieved 74% follow-up at the child age 13 years, despite the last previous assessment being at age 18 months. Based on this, we conservatively estimate that at least 80% of families recruited will be retained at follow-up, meaning that an initial cohort of 250 families is planned to ensure complete data for 200 families.

### Data analysis

Regression-based methods will be used to test the capacity of caregiver variables to predict child PTSD symptoms at 3 and 6 months after the traumatic event, controlling for the child’s initial symptoms and other potential confounders (eg, trauma severity, child age and gender, caregiver adjustment) (objective a). Patterns of missing data will be examined, and multiple imputation methods will be used if appropriate.^46^ We will test hypothesised indirect or mediated longitudinal pathways from caregiver mental health and responding to child outcome using bootstrapped estimates (objectives b). Objectives c and d will be addressed through descriptive statistics (PTSD diagnoses based on clinical interview, and through re-running the regression analyses conducted to address objective a, substituting internalising and externalising symptoms as the outcomes, with adjustment for co-occurring PTSS where appropriate, as per our previous work.^47^ Regression-based methods will be used to test whether cardiac biomarkers (heart rate and heart rate variability) predict child PTSD symptoms at 3 and 6 months after the traumatic event, controlling for the confounders assessed in objective a. Genome-wide DNA methylation levels will be extracted from DBS samples for future research purposes, including submission to epigenetic consortia for meta-analysis (objective e).

## Ethics and dissemination

### Ethics

Ethical approval was granted from Stellenbosch University, South Africa (reference no. N21/07/065) and the University of Bath, UK (reference no. 22-055.) The Sinethemba study is designed with child protection principles in mind, and the impact of the data collection process on participants is a foremost concern. The study will follow ethical procedures used by the researchers in other studies working with similar at-risk groups. Research staff are experienced in working with vulnerable children and will undergo refresher training before the implementation of the project. Prior to starting the interviews, caregivers will complete informed consent for themselves and for their child, and children will provide informed assent. Different versions of the informed consent and assent forms will be provided to caregivers, children aged 8–12 years, and children aged 13–16 years, in age-appropriate language. Participants will be assured that their decision to participate is voluntary and will have no impact on their medical care or legal rights. All versions of the information sheets, consent forms and questionnaires will be available in isiXhosa and English. Participants will be given the option to complete questionnaires independently or have the questions read aloud by the data collector who will capture responses. If the child’s preference is to complete the questionnaires independently, the data collector will first check that the child understands the questionnaire process by asking the participant to read the instructions and the first few questions aloud.

Participants will be able to withdraw from the study at any time. To ensure participants do not feel social pressure to participate, even after they have consented, the voluntary nature of the study is emphasised during the informed consent procedure, before and during the research assessments. Children and caregivers will be made aware they can choose to not participate in specific tasks. All information collected will remain strictly confidential within the research team, except in cases where there is high concern for the participant’s or someone else’s safety, as per standard research policy. Participants will be reassured that collection of biological data is optional, and children will be able to participate in the study even if they decline to take part in these assessments. The information sheet includes a statement to explain that biological data collection is for research purposes only and cannot be used to detect health issues.

While participants will be asked to only describe elements of the traumatic event they feel comfortable sharing, the questions will mean recollecting the event, which may cause distress or anxiety. Therefore, there will be specific policies in place to manage situations where a participant becomes distressed. A full risk protocol, including guidelines for responding to participant disclosure of significant psychological distress and documents to support referral to services where appropriate, is in place. Data collectors are trained and experienced in managing risk issues and the presentation of distress. Participants will be referred to the study case worker for assessment when necessary, which will lead to the offer of referral to support services where appropriate. This will include expressing distress, or specific answers to questions regarding mental health difficulties, substance misuse, domestic violence or food insecurity. Guidelines are also in place to protect the mental health of the research team. Data collectors will have weekly supervision sessions and are encouraged to disclose any difficulties, in addition to an open offer for one-to-one counselling.

Participants will be offered an R180 voucher at each time point. The amount provided reflects the time and effort taken to participate and is compliant with the standard reimbursements for research participation required by the Stellenbosch University Research Ethics Committee. The incentive amount is intended to be sufficient to ensure participants do not have financial loss through taking part, but not so high that participants will be motivated solely for financial gain.

### Dissemination

The results deriving from the Sinethemba study will examine social, psychological and biological influences on child experiences of trauma in contexts of ongoing high risk, an area with limited available evidence. In addition, this research will allow for a contribution to our theoretical understanding of the development of PTSD in such settings and may challenge existing assumptions about what comprises adaptive support and coping following child trauma that predominantly derives from HIC settings. Findings will be disseminated via publication in high-impact journals, workshops aimed at practitioners and researchers, and public engagement events in the community.

Findings will have a wide impact potential for public health. First, the majority of phenomenological data about trajectories of PTSD in the aftermath of child trauma derives from relatively low-risk populations predominantly exposed to relatively low-severity events. The Sinethemba study will provide evidence of how children in high-risk contexts respond and recover following trauma, which is critical to informing identification and intervention programmes. Second, to date, most interventions for child PTSD have been developed in HICs and thus may not translate well to contexts of ongoing adversity due to problems relating to conceptualisation and delivery. This research will also provide key information about the processes that contribute to recovery versus sustained PTSD in children in an LMIC setting, which can provide the foundation for intervention development or adaptation. We will maximise the impact of this study by (1) identifying and disseminating key potential intervention and policy points that can have real-world application; (2) developing plans for translating findings to clinical intervention approaches, if appropriate and (3) initiating further international projects to expand our understanding of children coping with trauma in a range of high adversity settings.
