# Supplementary material for: Post-traumatic stress disorder symptoms following exposure to acute psychological trauma in children aged 8–16 years in South Africa: protocol for the Sinethemba longitudinal study
Source: BMJ Open. 2024 Jul 11;14(7):e085129. doi: 10.1136/bmjopen-2024-085129 (PMC11624694; doi:10.1136/bmjopen-2024-085129)
Supplement: Supplementary file 1 [file bmjopen-2024-085129.reviewer_comments.pdf]

PEER REVIEW HISTORY

BMJ Open publishes all reviews undertaken for accepted manuscripts. Reviewers are asked to complete a checklist review form (**http://bmjopen.bmj.com/site/about/resources/checklist.pdf**) and are provided with free text boxes to elaborate on their assessment. These free text comments are reproduced below.

ARTICLE DETAILS

**TITLE (PROVISIONAL)**	Post-traumatic stress disorder symptoms following exposure to acute psychological trauma in children aged 8-16 years in South Africa: protocol for the Sinethemba longitudinal study
**AUTHORS**	Sharp, Tamsin; Chideya, Yeukai; Giuliani, Alessandra; Hunt, Xanthe; Tomlinson, Mark; Seedat, Soraya; Creswell, Cathy; Fearon, Pasco; Hamilton-Giachritsis, Catherine; Hiller, Rachel; Meiser-Stedman, Richard; Du Toit, Stefani; Stewart, Jackie; Halligan, Sarah

This article was not externally reviewed at BMJ Open. Protocols that have been independently assessed prior to submission to BMJ Open are usually fast-tracked to publication on the grounds that further substantial changes will not be possible. This independent assessment will usually be external, independent review for both a substantial grant award from a non-commercial or government funder and ethics approval.
